# Childhood cancer in Britain: incidence, survival and mortality

**DOI:** 10.1038/sj.bjc.6603800

**Published:** 2007-06-12

**Authors:** K Pritchard-Jones

**Affiliations:** 1 Institute of Cancer Research and Royal Marsden NHS Foundation Trust, London, UK.

Charles Stiller and colleagues at the Childhood Cancer Research Group (CCRG) in Oxford have published the first comprehensive overview of records held in the National Registry of Childhood Tumours (NRCT) since its inception. This is the longest established national population-based childhood cancer registry in the world. As of 2006, it includes nearly 80 000 cases, based on data collected prospectively since 1962 to the present day.

Contributions by Gerald Draper, who headed the CCRG from its inception through to 2002, describe the evolution of the Registry with its increasing completeness of case ascertainment and data quality. One of the strengths of the NRCT is the ability within the British National Health Service to ‘flag’ survivors so that the NRCT receives notification of their death or development of any subsequent cancer. This means that follow-up for mortality is virtually complete within Great Britain.

The NRCT has become an exemplar for establishing childhood cancer registries around the world. It is recognised to have extremely complete data, partly due to notification of cases from multiple sources, aided by the close relationship between the CCRG and the main childhood cancer clinical trials organisation in the UK (UK Children's Cancer Study Group, now known as the Children's Cancer and Leukaemia Group).

Owing to the long established high quality of case ascertainment, the NRCT is able to produce robust data on incidence of the main subtypes of childhood cancers and their changes in incidence over a 35-year period. These data confirm those seen across Europe and in some other Western populations that the overall incidence of childhood cancer is increasing by almost 1% per year over the last 30 years. The NRCT provides a valuable resource for further epidemiological research into why this should be. The ability to link records to information held on birth certification, such as parental occupation and extremely accurate location of birth due to the British postal code system, which can be linked to socio-economic status and environmental exposures, provides rich avenues for future research to investigate this phenomenon.

There is a detailed analysis of survival by cancer type and subtype for those treated in the most recent era for which 5-year survival are mature (i.e. 1991–2000). These are very valuable data for researchers interested in outcome according to biological basis of disease, due to the very detailed information collected by the CCRG, which is often not available to other cancer registries. These items include extra data on laterality for retinoblastoma and renal tumours and immunophenotyping for leukaemias and lymphomas. For cases diagnosed between 1991 and 2000, such data were complete for >95% and approximately 80% of cases, respectively.

Time trends in survival across successive quinquennia from 1966–1970 to 1996–2000 are also presented, showing encouraging and significant improvements for all diagnostic groups. The stubborn subgroups for which survival remains at less than 50% in the most recent period are highlighted.

The section on long-term survival for those who have achieved 5-year survival from original diagnosis, according to the quinquennium of their original diagnosis, will be of interest both to patients and to clinicians alike. It is reassuring to see that for the vast majority of childhood cancers, 5-year survival equates to an extremely good chance of much longer term survival, usually far in excess of 90%.

Finally, this book presents complete national data on mortality and trends in mortality by tumour type. These are essential to monitor the effectiveness of new therapeutic or preventative strategies. They also emphasise the important relative ranking of childhood cancer as a cause of death in children living in a modern Western society: over 20% of all deaths occurring in 1- to 14-year-olds are due to cancer and this relative proportion is increasing. This means that general child mortality is decreasing faster than childhood cancer mortality, emphasising that there remains much progress to be made.

The CCRG have lain before us their international wares and invite interested researchers to collaborate with them and maximise the opportunities for research and increasing the knowledge of childhood cancer aetiology and efficacy of treatment offered by their data collection. This book is an essential reading and a valuable reference source for all those responsible for organising, delivering and evaluating cancer care for children around the world.

